# Integrating Water Column and Sediment eDNA Metabarcoding Reveals Seasonal Turnover and Habitat‐Specific Differentiation of Fish Communities in a Subtropical Coastal Bay

**DOI:** 10.1002/ece3.73988

**Published:** 2026-07-09

**Authors:** Shannan Xu, Yayuan Xiao, Min Li, Jiangtao Fan, Zuozhi Chen

**Affiliations:** ^1^ South China Sea Fisheries Research Institute Chinese Academy of Fishery Sciences, Key Laboratory of Marine Ranching, Ministry of Agriculture and Rural Affairs Guangzhou P. R. China; ^2^ Guangdong Provincial Observation and Research Station for Ecosystem in the Pearl River Estuary Guangzhou P. R. China; ^3^ Observation and Research Station of Xisha Island Reef Fishery Ecosystem of Hainan Province Sanya P. R. China; ^4^ Key Laboratory for Sustainable Utilization of Open‐Sea Fishery Ministry of Agriculture and Rural Affairs Guangzhou P. R. China

**Keywords:** alpha and beta diversity, environmental DNA (eDNA), fish community structure, indicator species, spatiotemporal dynamics, subtropical coastal ecosystem, water–sediment interface

## Abstract

Environmental DNA (eDNA) metabarcoding provides a non‐invasive tool for monitoring marine biodiversity in complex ecosystems. In this study, we applied eDNA techniques to investigate the spatiotemporal dynamics of fish communities in Daya Bay, South China Sea, by analyzing 120 water column and 113 sediment samples collected across four seasons. Our research aimed to characterize the seasonal turnover and habitat‐specific partitioning of fish community structures. The results revealed that sediment matrices exhibited significantly higher alpha diversity compared to water samples. However, sediment showed lower community heterogeneity—quantified as within‐group Bray–Curtis dispersion—particularly during the warm seasons (spring and summer) compared with the water column. Conversely, during the cold seasons (autumn and winter), sediment matrices showed lower alpha diversity but higher community heterogeneity. Using LEfSe and random forest models, we identified a suite of discriminative indicator taxa that characterized these community shifts. Based on relative read abundance (RRA), *Johnius* and *Pennahia* were significantly enriched in the water column, while *Cynoglossus* and *Zebrias* were primarily associated with sediment habitats. These findings highlight the complementary roles of water and sediment eDNA in capturing the full spectrum of fish biodiversity. While this study primarily describes observed patterns within a dynamic hydrographic context, it provides foundational data for advancing eDNA‐based biomonitoring in subtropical marine ecosystems and supports the development of targeted conservation strategies.

## Graphical abstract

This study contributes foundational data for advancing eDNA‐based biomonitoring in subtropical marine ecosystems and supports the development of more targeted conservation and adaptive management strategies amid increasing anthropogenic pressures.

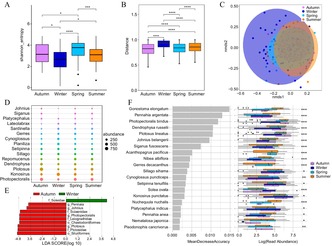

## 1 Introduction

Environmental DNA (eDNA) metabarcoding has revolutionized marine biodiversity monitoring by enabling the non‐invasive detection of fish communities across habitat boundaries and temporal scales (Le Joncour et al. 2024; Thomsen and Willerslev 2015). This approach addresses critical limitations of traditional trawl surveys, which may underestimate cryptic species and small‐bodied taxa in complex benthic‐pelagic ecosystems due to gear selectivity and constraints related to habitat accessibility (Le Joncour et al. 2024; Ip et al. 2024; Kasmi et al. 2024). By capturing trace genetic material from water and sediment matrices, eDNA techniques provide enhanced sensitivity for reconstructing species assemblages across seasonal cycles (Djurhuus et al. 2020). However, limited understanding persists regarding how habitat‐specific eDNA turnover dynamics interact with seasonal hydrographic forcing to shape observable biodiversity patterns (Joseph et al. 2022; Troth et al. 2021). These challenges may be particularly pronounced in tropical/subtropical bays (Du et al. 2021; Cabral et al. 2020), where strong seasonal stratification and rapid hydrological shifts—often exacerbated by anthropogenic stressors—could decouple real‐time biological signals in the water column from longer‐term eDNA archives in sediments (Collins et al. 2018; Schaeffer et al. 2023). However, in shallow coastal systems, these archives are increasingly recognized as dynamic reservoirs that integrate biological signals over relatively short temporal scales (e.g., weeks to months) compared to the ‘snapshot’ nature of water samples. Recent studies have increasingly emphasized the importance of temporal variation in eDNA‐based biodiversity assessments, particularly in marine and tropical ecosystems (Kaushik and Dixit 2024; Mathon et al. 2022; Bhendarkar and Rodriguez‐Ezpeleta 2024). These works highlight the seasonal turnover of community composition and the need for longitudinal monitoring to capture biodiversity trends accurately.

Daya Bay, a semi‐enclosed subtropical embayment in the South China Sea, epitomizes the challenges of monitoring biodiverse coastal ecosystems under anthropogenic pressures (Zhang et al. 2020; Wu et al. 2009). This ecologically significant region sustains a blend of tropical and subtropical fish communities, with small‐bodied benthic species increasingly dominating over the past three decades due to the impacts of intensive bottom trawling, eutrophication, and environmental contaminants (Zhang et al. 2020; Zhao, Chen, et al. 2024; Guo et al. 2008). Conventional monitoring approaches, particularly trawl surveys, may be limited in detecting these ecological transitions due to inherent gear selectivity biases and insufficient spatial resolution, resulting in an incomplete understanding of biodiversity dynamics (Ip et al. 2024). eDNA metabarcoding, especially when coupled with both water and sediment sampling, offers a robust method for overcoming these constraints by capturing real‐time biological activity in the water column and historical eDNA records in sediments (Ruppert et al. 2019). However, its application in subtropical bays such as Daya Bay may be complicated by pronounced seasonal hydrodynamics, including thermal stratification and rapid hydrological shifts. Seasonal variations may induce differences in the degradation patterns of eDNA in the water column and sediment. In the water column, elevated summer temperatures and UV exposure may accelerate eDNA decay (Strickler et al. 2015; Tsuji et al. 2017), whereas cooler winter temperatures may slow it (Eichmiller et al. 2016). In sediments, thermal buffering and anoxic microenvironments could attenuate seasonal fluctuations (Parducci et al. 2017; Pawlowski et al. 2022). These medium‐specific dynamics may contribute to notable spatiotemporal fluctuations in eDNA monitoring results. Furthermore, the decoupling of signals may be further exacerbated by urbanization‐driven changes in sediment organic loads (Guo et al. 2008; Zhang et al. 2018), resulting in systematic biases in cross‐habitat community analyses, particularly along the sediment‐water continuum (Cowart et al. 2022). These complexities highlight a significant gap in our understanding of how environmental changes influence eDNA monitoring, underscoring the need to enhance the accuracy and reliability of community assessments in tropical and subtropical ecosystems.

Although environmental stressors significantly influence coastal biodiversity, the foundational dynamics of fish eDNA at the sediment–water interface remain poorly understood. Here, we present a comprehensive spatiotemporal eDNA assessment of fish communities in Daya Bay. Recognizing the complexity of this ecosystem, our study focuses on characterizing observed community patterns and habitat partitioning rather than developing explicit predictive models for specific environmental drivers. By systematically comparing eDNA signals from both the water column and sediment habitats, we aim to elucidate potential discrepancies in community richness and composition across distinct temporal and spatial scales. This integrative approach highlights the complementary roles of different sampling matrices in capturing the full spectrum of fish biodiversity. Our findings provide essential baseline data to improve the reliability of eDNA‐based monitoring in subtropical coastal environments, particularly where complex hydrographic forcing creates highly dynamic biological signals.

## 2 Materials and Methods

### 2.1 Study Area

**FIGURE 1:**
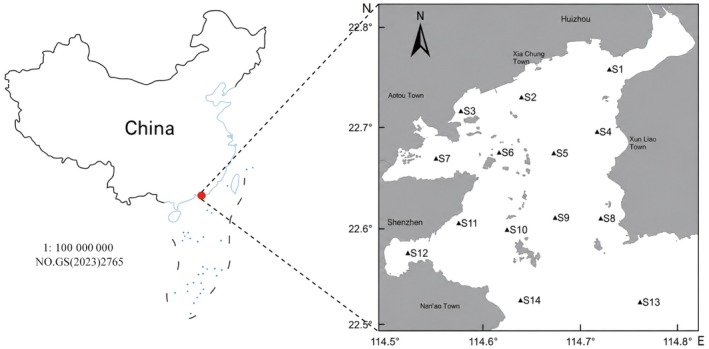
Map of sample collection sites in Daya Bay, South China Sea, eastern Guangdong Province, China.

The Daya Bay (22°30′–22°50′ N, 114°30′–114°50′ E) (Wang et al. 2011), a semi‐enclosed subtropical embayment in Guangdong Province, China (Figure 1), spans approximately 600 km^2^ within a mountainous coastal landscape bounded by Huiyang District (north), Shenzhen City (west), Huidong County (east), and the South China Sea (south). Characterized by diverse inshore habitats including island‐fringed reefs, sheltered coves, and estuarine zones, this ecosystem maintains a mean annual temperature of 21.8°C. It serves as critical spawning/nursery grounds for many fish species, supporting commercially important stocks in the South China Sea coastal fisheries. Significant seasonal shifts characterize the hydrographic regime of Daya Bay. During the summer (typically April to September), strong thermal stratification develops, with surface water temperatures often reaching 30°C, resulting in a distinct thermocline (Wu et al. 2009; Wang et al. 2008). In contrast, the winter season is dominated by the northeast monsoon, which facilitates intense vertical mixing and potential resuspension of surface sediments (Wang et al. 2011). Additionally, the bay's hydrology is influenced by the intrusion of the South China Sea Surface Current and local freshwater discharge, creating complex spatiotemporal gradients in salinity and dissolved oxygen that potentially shape the distribution and detectability of fish eDNA (Zhang et al. 2020; Wang et al. 2008).

Designated as a provincial nature reserve in 1983 and subsequently prioritized for economic development in 1984, the bay has experienced escalating anthropogenic pressures. Intensified bottom trawling, expansion of mariculture operations, and coastal industrialization (nuclear power plants, petrochemical complexes, and port facilities) have cumulatively driven the fisheries ecosystem degradation. Long‐term monitoring data have revealed a pronounced decline in fishery biodiversity since the early 1990s (Zhao, Chen, et al. 2024), along with significant alterations in fish population structure and a shift in community composition toward small‐bodied benthic dominance.

### 2.2 Sample Collection, Filtration, and Preservation

Four seasonal oceanographic surveys were conducted in Daya Bay in October 2022 (autumn), January 2023 (winter), April 2023 (spring), and August 2023 (summer) (Figure 1). All sampling and field operations were carried out in strict accordance with standardized national protocols, including the Marine Monitoring Specification (GB 17378‐2007), Marine Survey Specification (GB/T 12763‐2007), and Coastal Water Environmental Monitoring Specification (HJ 442‐2008).

A total of 14 fixed sampling stations were established across the bay. Daya Bay is a typical subtropical semi‐enclosed embayment in the northern South China Sea, spanning approximately 650 km^2^ with an average depth of 11 m. It exhibits weak hydrodynamic conditions with small tidal ranges and low wave energy. The station layout considered the bay's geomorphological enclosure, habitat heterogeneity, and hydrological zoning. Referring to previous regional ecological studies (Wang et al. 2008; Zhang et al. 2020; Xiang et al. 2021; Shi et al. 2025), it is well established that 10–20 sites are sufficient to capture spatial biodiversity variation in such systems. Accordingly, 14 representative stations were selected to encompass the bay head, central basin, mouth, and adjacent offshore zones. This configuration aligns with the long‐term ecological monitoring framework used in Daya Bay between 2014 and 2023, facilitating historical comparisons and trend analysis.

In this study, 5 m was chosen as the threshold for stratified water sampling because it approximates half of the bay's mean depth. At sites deeper than 5 m, stratified water sampling was conducted by collecting separate subsamples from the surface, midwater, and bottom layers, which were subsequently pooled in equal volumes for filtration. This pooling approach was strategically adopted with the primary objective of maximizing species detection at each sampling station and ensuring a comprehensive assessment of the total fish biodiversity across the entire water column. Given that Daya Bay is a relatively shallow coastal system characterized by frequent tidal mixing and wave action, a composite sample from multiple depths provides a more integrated profile of the local fish community. While we acknowledge that this strategy represents a composite signal of the water column and may blur fine‐scale vertical structuring—a feature that can be particularly relevant during periods of seasonal stratification—it was prioritized in this study to enhance detection sensitivity for broader spatiotemporal and habitat‐specific comparisons. In future marine eDNA studies, whether to use stratified sampling—and how many layers to include—should be determined based on site‐specific factors such as water depth, hydrography, and research objectives.

The water samples were filtered using a diaphragm vacuum pump (Huankai, China) connected to a vacuum filtration apparatus equipped with 0.45 μm Mixed Cellulose Ester (MCE) membrane filters (Whatman, UK). A total of 5 L of seawater (either pooled from stratified layers or from the surface) was filtered at each station. After filtration, membranes were carefully removed using sterile forceps, placed into sterile cryotubes, and immediately flash‐frozen in liquid nitrogen. All filtration units, tubing, forceps, and containers were sterilized in 10% sodium hypochlorite solution for 30 min and then rinsed thoroughly three times with sterile distilled water. Field personnel wore disposable gloves and changed them between stations to avoid cross‐contamination.

For sediment sampling, approximately 50 mL of surface sediment was collected per replicate using sterile 50 mL centrifuge tubes. Triplicate sediment samples were taken at each station. The tubes were sealed, flash‐frozen in liquid nitrogen on board, and transferred under cold chain to the laboratory, where they were stored at −80°C until DNA extraction. The sampling scheme was rigorously structured to ensure comprehensive spatial and temporal coverage. Across four seasons and 14 stations, two sample types (filtered seawater and sediment) were collected with three biological replicates each, yielding a theoretical total of 336 samples. Complete sample metadata are provided in Table S1.

### 2.3 Genomic DNA Extraction and Quality Control

Total genomic DNA was extracted from both water and sediment samples using the FastLee DNA Spin Kit for Feces (FastBio, China) according to the manufacturer's instructions, with minor adjustments to optimize yield and consistency for environmental samples. For water samples, each filter membrane was aseptically cut into small strips (~0.5 × 0.5 cm) using sterile scissors and transferred into a 2 mL bead‐beating tube pre‐loaded with ceramic beads. For sediment samples, 0.25 g of homogenized wet sediment was measured and transferred into the same type of extraction tube. Mechanical lysis was performed using a Mini‐Beadbeater‐16 (BioSpec, USA) at 3500 rpm for 45 s to ensure effective cell disruption. This was followed by chemical lysis with the kit's lysis buffer and proteinase K at 65°C for 10 min, with intermittent vortexing. After lysis, total DNA was isolated through silica column purification steps according to the kit protocol. To monitor for potential cross‐contamination during the extraction process, an extraction blank (nuclease‐free water) was included in parallel with each batch of samples. All extractions were conducted in a dedicated clean‐room laboratory, and pre‐PCR and post‐PCR processes were spatially separated to minimize contamination risk.

The quality and quantity of extracted DNA were assessed using two complementary methods. First, 1% agarose gel electrophoresis, stained with SYBR Safe DNA Gel Stain (Invitrogen), was used to confirm DNA integrity. Second, spectrophotometric analysis was performed with a NanoDrop ND‐2000 (Thermo Fisher Scientific, USA) to measure DNA concentration (A260) and purity ratios (A260/A280 and A260/A230). DNA samples with A260/A280 ratios between 1.7 and 2.0 were considered acceptable. All DNA samples were subsequently diluted to a working concentration of 1 ng/μL using nuclease‐free water. Aliquots were stored in DNA LoBind tubes (Eppendorf) at −20°C for short‐term use, and long‐term backups were stored at −80°C until further processing for PCR and sequencing.

### 2.4 12S rRNA Gene Amplification and Amplicon Validation

To characterize fish community composition, the mitochondrial 12S rRNA gene was amplified using MiFish‐U primers (Miya et al. 2015) (Forward: 5′‐GTYGGTAAAWCTCGTGCCAGC‐3′; Reverse: 5′‐CATAGTRGGGTATCTAATCCYAGTTTG‐3′), each tagged with unique barcodes for sample identification. PCR reactions were performed in a 30 μL volume, containing 15 μL of Phusion High‐Fidelity PCR Master Mix (New England Biolabs, USA), 0.2 μM of each primer, and approximately 10 ng of template DNA. Amplification was carried out on a Bio‐Rad C1000 thermocycler with the following conditions: initial denaturation at 98°C for 1 min; 30 cycles of denaturation at 98°C for 10 s, annealing at 50°C for 30 s, and extension at 72°C for 60 s; followed by a final extension at 72°C for 5 min. To minimize potential PCR inhibition, which is commonly associated with environmental samples, all DNA templates were standardized to 1 ng/μL prior to amplification. While per‐sample inhibition testing was not performed, this uniform dilution strategy was employed to reduce inhibitor concentrations to negligible levels for the high‐fidelity polymerase.

To assess potential laboratory‐based contamination, a set of three negative controls—consisting of DNA extraction blanks (nuclease‐free water processed alongside the samples) and PCR no‐template controls (NTCs)—was used in each seasonal batch, totaling 12 blank samples for the entire study. These controls were processed with the same MiFish primers and conditions as the environmental samples. Importantly, gel electrophoresis results showed no visible amplification products from any of these negative controls, indicating an absence of significant systematic contamination during the laboratory workflow. As no visible amplification was observed, these negative controls were not subjected to high‐throughput sequencing, and thus no bioinformatic sequence removal or thresholding was required. While field and filtration blanks were not implemented in this study, the lack of amplification in laboratory blanks, combined with the high consistency of community profiles across biological replicates, supports the overall reliability of the reported fish eDNA signals. Environmental sample amplicons showing a single distinct band of approximately 170 bp were retained for further processing. Qualified products were normalized to equimolar concentrations, pooled, and purified using the GeneJET Gel Extraction Kit (Thermo Scientific, USA) according to the manufacturer's protocol.

### 2.5 Library Preparation, Sequencing, and Quality Control

Sequencing libraries were constructed with the NEBNext Ultra DNA Library Prep Kit for Illumina (New England Biolabs, USA) following the supplier's recommendations, and unique index adapters were ligated. Library size distribution and concentration were verified using an Agilent 2100 Bioanalyzer (Agilent Technologies, USA) and a Qubit 2.0 Fluorometer (Thermo Fisher Scientific, USA). Paired‐end sequencing (2 × 250 bp) was performed on an Illumina novaseq platform (Illumina, USA).

High‐throughput sequencing of the amplicon libraries was conducted, and sequences containing a high proportion of non‐fish reads were removed during quality control. After the initial quality filtering and denoising, taxonomic assignment was performed for all resulting ASVs. Non‐fish sequences were then identified and removed using a taxonomic filtering approach, where only ASVs assigned to the Superclass Actinopterygii and Class Chondrichthyes were retained for downstream analysis. Following the removal of these non‐target ASVs (e.g., those assigned to humans, mammals, or unassigned reads), we further applied a read‐depth threshold to ensure data reliability. Specifically, samples with fewer than 5004 sequence reads were excluded from downstream analyses. As a result, out of the 336 initially processed samples, 233 samples with sufficient sequencing depth were retained for community analysis. Despite this attrition, the retained samples (averaging > 28 samples per season/habitat) ensured comprehensive coverage across all 14 stations and four seasons. We prioritized this stringent filtering to ensure that ecological inferences were based on high‐quality sequence data, thereby minimizing potential biases introduced by samples with insufficient read depth.

### 2.6 Bioinformatics Data Processing and Analysis

All bioinformatic analyses were conducted in QIIME 2 (version 2022.2) following the official tutorials with minor adaptations (https://docs.qiime2.org/2022.2/tutorials/). Briefly, raw paired‐end reads were demultiplexed and subsequently subjected to quality filtering, denoising, paired‐end merging, and chimera removal using the DADA2 plugin (Callahan et al. 2016). Resulting non‐singleton amplicon sequence variants (ASVs) were multiple‐sequence aligned with MAFFT (Katoh et al. 2019).

To standardize sampling effort, feature tables were rarefied to 5004 sequences per sample before diversity estimation. Alpha‐diversity metric (Shannon index) was calculated using QIIME 2's diversity plugin. Beta‐diversity was assessed via non‐phylogenetic (Bray–Curtis dissimilarity) metrics. Taxonomic assignment of ASVs was performed using the classify‐sklearn naïve Bayes classifier trained against a custom‐built, non‐redundant fish mitochondrial reference database (Abraham et al. 2014). This database was constructed by integrating fish mitochondrial sequences from both MitoFish and the NCBI GenBank public database. During the construction process, we implemented strict filtering criteria to remove sequences with ambiguous taxonomic information or those that were excessively short. To ensure the efficiency and accuracy of the classifier, the sequence set was de‐replicated at a 99% similarity threshold to generate a comprehensive non‐redundant reference set. This curated database includes mitochondrial 12S rRNA gene sequences from diverse geographical regions, including regional fish species specific to the South China Sea, thereby ensuring high taxonomic resolution for the Daya Bay fish community.

For ordination analyses, non‐metric multidimensional scaling (NMDS) was performed using the Bray–Curtis distance matrix computed above. NMDS was run with 999 random starts in QIIME 2's diversity plugin, and the two‐dimensional solution was retained for visualization. The resulting NMDS plot displays sample relationships in reduced dimensional space, where inter‐sample distances reflect compositional (taxonomic) dissimilarities.

To identify taxa that discriminate between experimental groups, we performed Linear Discriminant Analysis Effect Size (LEfSe) (Segata et al. 2011) on the relative‐abundance data. LEfSe is specifically designed for high‐dimensional microbiome datasets and combines statistical significance with effect‐size estimation to detect robust biomarkers (LDA Score > 3, *p* < 0.05). In addition, we employed Random Forest classification to further evaluate group separation and determine the contribution of individual taxa to model performance. Random Forest is an ensemble learning algorithm that constructs multiple decision trees on bootstrap‐resampled subsets of the data and aggregates their votes for final prediction, making it suitable for both classification and regression tasks. Importantly, the algorithm computes variable importance scores—typically quantified by mean decrease in accuracy and mean decrease in Gini impurity—which provide a ranked assessment of each taxon's influence on classification accuracy (Breiman 2001).

## 3 Result

### 3.1 Ecological Differentiation of Fish Communities Between Water Column and Sediment Habitats in the Daya Bay

**FIGURE 2:**
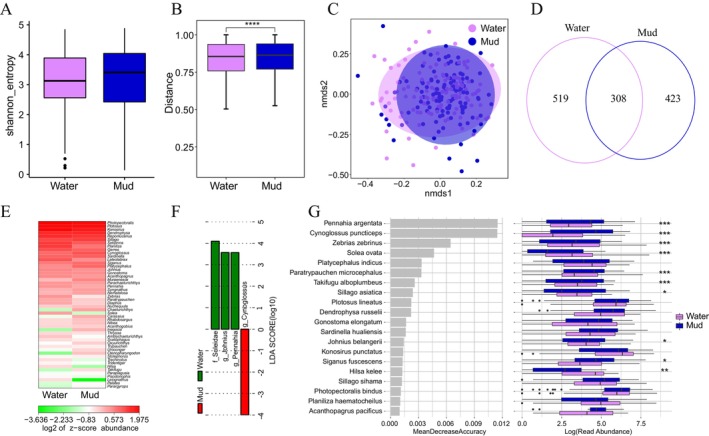
Comparative analysis of fish communities between the water column and sediment habitats across all samples (*n* = 233) in Daya Bay. (A) Alpha diversity (Shannon index, Mann–Whitney *U* test) comparison between different habitats. (B) Bray‐Curtis‐based beta diversity showing community heterogeneity. (C) NMDS analysis revealed differences in community structure. (D) Venn diagram showing ASV overlap between water and sediment samples. (E) The top 50 highly detected fish genera in each habitat. (F) LEfSe analysis identifying significantly enriched taxa in different habitats (LDA > 3, *p* < 0.05). (G) Random forest model showing top‐ranking taxa contributing to habitat differentiation. Water: The water column group. Mud: The sediment mud group. *p < 0.05, **p < 0.01, ***p < 0.001, ****p < 0.0001.

Using eDNA metabarcoding, this study systematically characterized the structural differences in fish communities between the water column and sediment habitats of Daya Bay, South China Sea. All samples (*n* = 233) were categorized into two groups: the water column group and the sediment mud group. The Shannon diversity index revealed that alpha diversity was significantly higher in the sediment group than in the water group (Figure 2A). Furthermore, intergroup heterogeneity based on Bray–Curtis dissimilarity also indicated greater beta diversity in sediment samples (Figure 2B). However, non‐metric multidimensional scaling (NMDS) did not reveal a clear separation of global community structures, suggesting that the two habitats share a high proportion of core fish taxa (Figure 2C). A Venn diagram analysis identified 308 shared amplicon sequence variants (ASVs) between the water and sediment groups (Figure 2D), suggesting a high degree of species overlap across habitats. At the genus level, *Photopectoralis*, *Plotosus*, and *Konosirus* were highly detected taxa in both habitats, consistently exhibiting high RRA (Figure 2E). Despite the high degree of shared species, LEfSe analysis identified significant taxonomic differentiation through habitat‐specific biomarkers. *Johnius* and *Pennahia* were significantly enriched in the water group, while *Cynoglossus* was more abundant in the sediment group (Figure 2F). The random forest model also identified key species contributing to habitat differentiation (Figure 2G). Specifically, *Pennahia argentata* , *Cynoglossus puncticeps* , *Zebrias zebrinus* , *Paratrypauchen* microcephalus, and *Takifugu alboplumbeus* were strongly associated with sediment samples, whereas *Solea ovata* and *Sillago asiatica* were read‐enriched taxa in the water column group.

### 3.2 Spatiotemporal Heterogeneity of Habitat‐Specific Fish Communities Driven by Seasonal Variation

**FIGURE 3:**
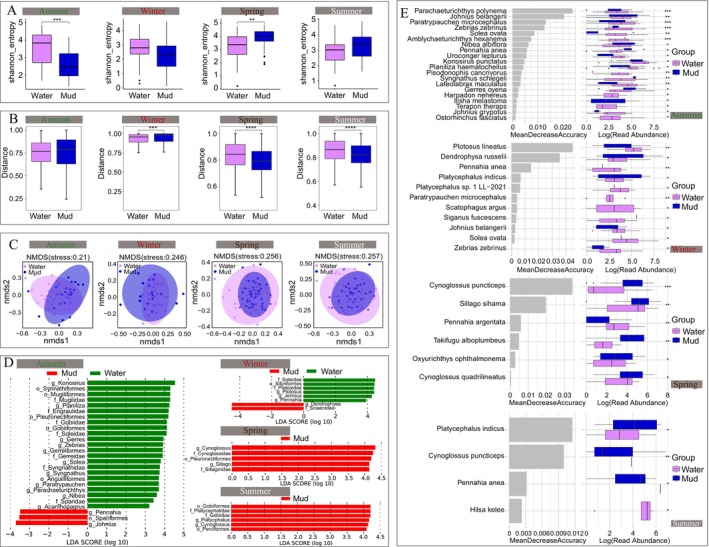
Seasonal variation in fish community composition and differentiation between water column and sediment habitats. (A) Seasonal shifts in Shannon alpha diversity between habitats (Mann–Whitney *U* test). (B) Seasonal beta diversity patterns using within‐group Bray–Curtis dissimilarity. (C) NMDS analysis showed seasonal differences between habitats (D). LEfSe analysis of seasonal indicator taxa by habitat. (E) Random forest model identifying key indicator species associated with each season‐habitat combination. Water: The water column group. Mud: The sediment mud group. *p < 0.05, **p < 0.01, ***p < 0.001, ****p < 0.0001.

To elucidate the effects of seasonal factors on fish community differentiation between water column and sediment habitats, we performed stratified spatiotemporal analyses using eDNA data from four seasonal sampling campaigns spanning autumn‐winter 2022 and spring–summer 2023. Alpha diversity exhibited contrasting seasonal patterns: sediment communities displayed significantly higher Shannon indices than water column assemblages during spring and summer, while this relationship reversed in autumn and winter (Figure 3A). Beta diversity analysis further revealed seasonal heterogeneity, with water column communities exhibiting significantly greater within‐group dispersion than sediment counterparts during spring–summer (Figure 3B). This pattern was corroborated by NMDS ordination, which demonstrated stronger structural overlap between habitat types in warm seasons compared to cold seasons (Figure 3C).

Integrated LEfSe and random forest analyses identified key seasonal indicator taxa in both habitats (Figure 3D, Figure S1). *Planiliza*, *Solea*, *Paratrypauchen*, and *Parachaeturichthys* were read‐enriched indicator genera in the water column, whereas *Pennahia* emerged as the read‐enriched indicator genus in sediment samples. During winter, the water community was primarily dominated by *Plotosus* and *Johnius*, while *Dendrophysa* was the characteristic genus in the sediment. Interestingly, no shared indicator taxa were detected in the water column for spring and summer, while *Cynoglossus* and *Sillago* were significantly enriched in spring sediment samples, and *Platycephalus* dominated the summer sediment assemblages.

These seasonal distribution patterns may reflect the habitat‐specific functional preferences of different fish taxa in response to season‐habitat combinations. Random forest modeling (Figure 3E) was further used to quantify the key indicator species contributing to habitat differentiation in each season. Autumn exhibited the highest number of discriminative taxa: 12 fish species were significantly enriched in water samples, including *Parachaeturichthys polynema* , *Paratrypauchen microcephalus*, *Solea ovata* , *Uroconger lepturus* , *Konosirus punctatus* , *Planiliza haematocheilus*, *Pisodonophis cancrivorus* , *Lateolabrax maculatus*, *Harpadon nehereus* , *Terapon theraps* , *Johnius grypotus* , and *Ostorhinchus fasciatus*. Meanwhile, 7 species were significantly enriched in sediment samples, including *Johnius belangerii* , *Zebrias zebrinus* , *Nibea albiflora* , *Pennahia anea* , *Syngnathus schlegeli* , *Gerres oyena* , and *Ilisha melastoma* . In winter, the water column was characterized by eight indicator species: *Plotosus lineatus* , *Pennahia anea* , *Platycephalus* sp. *1 LL−2021*, *Paratrypauchen microcephalus*, *Scatophagus argus* , *Johnius belangerii* , *Solea ovata* , and *Zebrias zebrinus* . The sediment community was primarily dominated by *Dendrophysa russelii* , *Platycephalus indicus* , and *Siganus fuscescens* . Although only six indicator taxa were identified in spring, they displayed clear habitat specificity: *Pennahia argentata* was enriched exclusively in the water column, while the sediment was dominated by *Cynoglossus puncticeps* , *Sillago sihama* , *Takifugu alboplumbeus* , *Oxyurichthys ophthalmonema* , and *Cynoglossus quadrilineatus* . In summer, water samples were characterized by *Cynoglossus puncticeps* , *Hilsa kelee* , and *Pennahia anea* , whereas *Platycephalus indicus* served as the read‐enriched indicator species in sediment samples.

### 3.3 Seasonal Succession Patterns of Fish Communities in Water Column and Sediment Habitats

The above results demonstrated that seasonal environmental fluctuations dynamically regulate fish community differentiation between water and sediment habitats. However, the specific response patterns of each habitat type to seasonal variations remain poorly characterized. To elucidate habitat‐specific seasonal adaptation strategies, we independently divided water column (*n* = 120) and sediment (*n* = 113) samples into four seasonal groups and systematically compared their spatiotemporal community dynamics.

**FIGURE 4:**
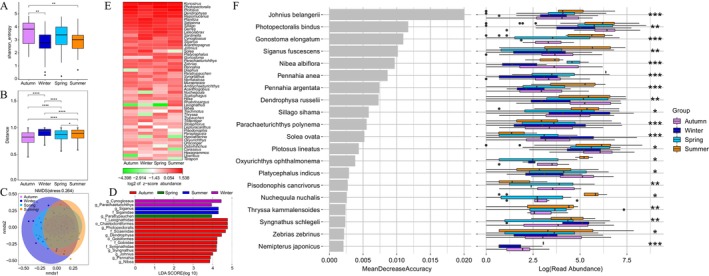
Seasonal succession patterns of water column fish communities (*n* = 120). (A) Seasonal alpha diversity (Shannon index, Mann–Whitney *U* test). (B) Seasonal beta diversity is based on Bray–Curtis's dissimilarity. (C) NMDS analysis revealed seasonal differences in fish community structure in the water column (D). The top 50 read‐enriched fish genera across seasons. (E) LEfSe‐identified seasonal indicator genera in the water column. (F) Random forest model identifying key seasonal indicator species. *p < 0.05, **p < 0.01, ***p < 0.001, ****p < 0.0001.

For the 120 water samples, Shannon diversity index analysis revealed that aquatic communities exhibited the highest diversity in autumn and the lowest in winter (Figure 4A). Within‐group heterogeneity based on Bray–Curtis dissimilarity was highest in winter (Figure 4B; Kruskal–Wallis test, *p* < 0.05), a finding corroborated by NMDS, which showed maximum community dispersion during the cold season (Figure 4C). At the genus level, *Konosirus* remained a stable, high RRA core taxon year‐round in the water column (Figure 4D). Comparative analysis revealed that winter aquatic communities manifested a discernible transitional pattern compared to the other three seasons, characterized by decreased RRA of *Photopectoralis*, *Dendrophysa*, and *Repomucenus*, alongside increases in *Planiliza*, *Sillago*, and *Cynoglossus*. LEfSe analysis further identified seasonally enriched taxa: *Cynoglossus* and *Parachaeturichthys* in winter; *Photopectoralis*, *Dendrophysa*, *Syngnathus*, *Johnius*, *Pennahia*, and *Nibea* in autumn; *Paratrypauchen* in spring; and *Siganus* in summer (Figure 4E). The random forest model further identified the key indicator species and their relative contributions for each season (Figure 4F). In spring, seven taxa— *Johnius belangerii* , *Nibea albiflora* , *Pennahia anea* , *Parachaeturichthys polynema* , *Pisodonophis cancrivorus* , *Zebrias zebrinus* , and *Nemipterus japonicus* —were the strongest discriminators of water column communities. Summer was characterized by nine indicator species, including *Gonostoma elongatum* , *Siganus fuscescens* , *Pennahia argentata* , *Dendrophysa russelii* , *Sillago sihama* , *Plotosus lineatus* , *Oxyurichthys ophthalmonema* , *Nuchequula nuchalis*, and *Thryssa kammalensoides* . In contrast, the number of key indicators dropped to just two in both autumn (*Photopectoralis bindus* and *Platycephalus indicus* ) and winter ( *Syngnathus schlegeli* and *Solea ovata* ), likely reflecting niche contraction and intensified environmental filtering under colder conditions.

**FIGURE 5:**
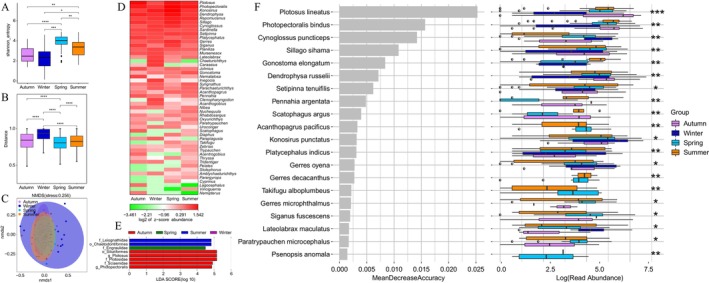
Seasonal succession patterns of sediment‐associated fish communities (*n* = 113). (A) Seasonal alpha diversity trends (Mann–Whitney *U* test). (B) Seasonal beta diversity by Bray–Curtis dissimilarity. (C) NMDS analysis revealed seasonal differences in fish community structure in the sediment habitat. (D) Heatmap of the top 50 read‐enriched genera across seasons. (E) LEfSe analysis of seasonally enriched sediment taxa. (F) Random forest results identifying seasonal indicator species in sediment samples. *p < 0.05, **p < 0.01, ***p < 0.001, ****p < 0.0001.

Seasonal grouping of the 113 sediment samples revealed distinct successional patterns. Alpha diversity declined from spring to winter (Figure 5A). Bray‐Curtis dispersal was greatest in winter (Figure 5B; Kruskal–Wallis test, *p* < 0.05), while NMDS indicated relatively low separation among seasons overall, with winter showing the greatest community spread (Figure 5C). At the genus level, *Photopectoralis* and *Dendrophysa* constituted stable core taxa across all seasons (Figure 5D). *Plotosus* and *Photopectoralis* were most enriched in autumn (Figure 5E), while *Konosirus* exhibited a marked RRA increase in winter. In spring and summer, the top five genera—*Plotosus*, *Photopectoralis*, *Konosirus*, *Dendrophysa*, and *Repomucenus*—maintained higher RRA, demonstrating broad warm‐season tolerance. Random forest analysis further quantified seasonal indicator species in sediments (Figure 5F): spring was marked by five species ( *Setipinna tenuifilis* , *Acanthopagrus pacificus*, *Takifugu alboplumbeus* , *Gerres microphthalmus* , *Paratrypauchen microcephalus*); summer by four species ( *Sillago sihama* , *Gonostoma elongatum* , *Platycephalus indicus* , and *Gerres decacanthus* ); autumn by four species ( *Plotosus lineatus* , *Photopectoralis bindus*, *Dendrophysa russelii* , and *Scatophagus argus* ); and winter by six species (Cynoglossus *puncticeps* , *Pennahia argentata* , *Konosirus punctatus* , *Gerres oyena* , *Siganus fuscescens* , and *Lateolabrax maculatus*).

### 3.4 Integrated Spatiotemporal Dynamics and Indicator Richness Contraction

**FIGURE 6:**
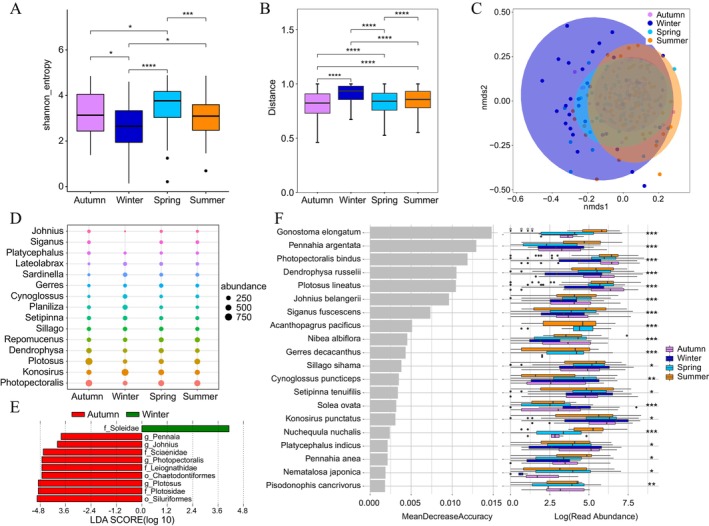
Integrative analysis of seasonal dynamics in fish communities across water column and sediment habitats (*n* = 233). (A) Seasonal variation in alpha diversity (Mann–Whitney *U* test). (B) Within‐group beta diversity across seasons. (C) NMDS plot illustrating seasonal dispersion of fish communities. (D) Top 15 read‐enriched fish genera in different seasons. (E) LEfSe‐derived seasonal indicator taxa across all samples. (F) Random forest model highlighting key seasonal indicator species. *p < 0.05, **p < 0.01, ***p < 0.001, ****p < 0.0001.

The integrated analysis of all water column and sediment samples (*n* = 233) reveals significant seasonal dynamics that characterize the fish community structure in Daya Bay. While the general trends in alpha and beta diversity (Figure 6A,B) echo the findings in individual habitats, the synthesized view emphasizes the higher dispersion of winter samples (Figure 6C). This maximal community spread in winter suggests that colder conditions may act as a period of intensified community restructuring.

Genus‐level analysis identifies two distinct ecological components: a cross‐seasonally stable component and a seasonally specific component (Figure 6D). The stable component comprises five core genera—*Photopectoralis, Konosirus, Plotosus, Dendrophysa*, and *Repomucenus*—which consistently rank among the top taxa in RRA year‐round. These genera demonstrate high ecological adaptability, maintaining a persistent presence across varying hydrographic regimes.

In contrast, the seasonally specific component reveals a pronounced contraction of indicator species richness during the cold period (Figure 6E,F). While the spring assemblage is characterized by seven discriminative species (e.g., *Johnius belangerii, Nibea albiflora*) and the summer community comprises nine characteristic indicators (e.g., *Gonostoma elongatum, Siganus fuscescens*), the number of robust indicator taxa drops significantly during the colder months. Only three indicator species are identified for both autumn (*Dendrophysa russelii, Plotosus lineatus, Platycephalus indicus*) and winter (*Setipinna tenuifilis, Solea ovata, Konosirus punctatus*) (Figure 6F). This reduction in indicator richness likely reflects a pronounced ecological niche contraction and intensified environmental filtering under lower temperature regimes. By integrating water and sediment data, these macro‐ecological patterns of seasonal turnover and community specialization become more clearly discernible.

## 4 Discussion

This study systematically investigated spatiotemporal partitioning of fish communities between water column and benthic habitats in Daya Bay, South China Sea, using eDNA metabarcoding. Integrated community composition analysis of 233 samples revealed profound combined effects of habitat type and seasonal variation on fish diversity patterns, assemblage structure, and ecological adaptation strategies, which could provide baseline data for tropical bay ecosystem monitoring and resource management. As a representative tropical–subtropical transitional bay in the northern South China Sea, Daya Bay exhibits characteristic warm‐water fish dominance (Zhang et al. 2020). In recent years, anthropogenic disturbances such as overfishing and fishing gear modernization have significantly reshaped the structure of regional fish communities (Zhang et al. 2020; Yu et al. 2022). Historical monitoring records have documented structural shifts in fishery resources: community transition from pelagic to demersal/benthic dominance, and phenotypic shift from large‐bodied commercial species to small‐sized low‐value taxa (Zhang et al. 2020; Zhao, Chen, et al. 2024; Zhao, Wang, et al. 2024). Our eDNA‐based findings suggest patterns consistent with previously reported ecological succession, indicating contemporary assemblages dominated by small‐sized, warm‐water, near‐bottom and benthic species. While these findings align with historical descriptions, we acknowledge that direct comparisons between eDNA‐based and traditional survey methods may be limited due to methodological differences and uncertainties in quantitative interpretation.

Our works have demonstrated that sediment habitats support significantly higher alpha diversity and greater community heterogeneity than the overlying water column, likely reflecting their complex microhabitats and elevated organic matter content (Pawlowski et al. 2022). As a “DNA reservoir,” sediment adsorbs particulate matter and, under low‐temperature, low‐oxygen conditions, slows eDNA degradation (Muschick et al. 2023; Can Yilmaz and Barnes 2025). In shallow coastal systems like Daya Bay, where sediment–water exchange is active, these sedimentary archives likely reflect short‐term integrated biological signals rather than long‐term historical records, allowing for the detection of seasonal community shifts within the benthic matrix. This capacity allows the accumulation of historical DNA signals over extended periods, thereby capturing species presence across multiple seasons or even years (Parducci et al. 2017; Barrenechea Angeles et al. 2020; Pedersen et al. 2015). For instance, in this study, the pronounced enrichment of the benthic flatfish Cynoglossus highlights its specialized adaptation to sediment habitats, and the year‐round detection of Photopectoralis may reflect that continuous metabolic activity maintains its eDNA signal in sediments. By contrast, the high hydrodynamic fluxes, temperature fluctuations, and UV exposure characteristic of the water column could accelerate DNA decay (Strickler et al. 2015; Tsuji et al. 2017; Eichmiller et al. 2016; Nukazawa et al. 2018), resulting in predominantly real‐time eDNA signals that may underestimate rare or transient taxa (Duarte et al. 2023; Chen et al. 2024). This dynamic partially accounts for the seasonal variation in eDNA detection efficiency (Troth et al. 2021; Buxton et al. 2018) for mid‐ to upper‐water species (e.g., *Johnius* and *Pennahia* spp.), where hydrodynamic dispersion likely dilutes their genetic signals, resulting in consistently low detectability except during autumn spawning aggregations when biomass concentration peaks (Deng et al. 2024; Zhang et al. 2019; Yan et al. 2011).

Although NMDS analysis failed to reveal significant separation between water and sediment communities, both LEfSe and random forest analyses identified representative taxa indicative of clear niche differentiation. Sediment‐enriched indicator species, such as *Cynoglossus puncticeps* and *Zebrias zebrinus* , are primarily benthic ambush predators that are adapted to a partially buried lifestyle in soft substrates (Karna et al. 2018; Kishipour et al. 2023; Wang et al. 2013). In contrast, water column‐dominant species, including *Sillago asiatica* and *Hilsa kelee* , exhibit broader feeding behaviors and typically occupy nearshore or mid‐to‐upper water layers (Dwivedi 2020; Xiao et al. 2016). Such functional group differentiation was often closely coupled with the physicochemical characteristics of the habitats (Dai et al. 2021; Geraldi et al. 2024; Liang et al. 2025): the heterogeneous sediment microenvironments could provide refugia for habitat‐specialist species, whereas the relatively homogeneous water column favors generalist species with greater dispersal capacity. Moreover, a considerable proportion of ASVs were shared between the two habitats (Figure 2D), indicating that certain species may exhibit strong cross‐habitat mobility or flexible habitat‐use strategies (Sánchez‐Núñez et al. 2024; Maciel et al. 2024; Reis‐Filho et al. 2016). Given that eDNA signal strength is affected by retention and degradation dynamics in the sampling matrix, integrating both water and sediment sampling enables a more balanced assessment of both real‐time and legacy biodiversity signals (Troth et al. 2021; Jo and Minamoto 2021; Shin et al. 2019). This integrative approach may help to reduce taxonomic biases associated with single‐substrate sampling and thus provides a more comprehensive and reliable framework for interpreting the spatiotemporal dynamics of fish communities in complex coastal ecosystems (Pawlowski et al. 2022; Shin et al. 2019; Brandt et al. 2021).

Seasonal analysis further revealed the complex spatiotemporal dynamics of fish community differences between the water column and sediment habitats. In the South China Sea, summer community structure was primarily driven by water environmental variables such as nutrient concentrations and surface water temperature, whereas in winter, geographical factors (e.g., latitude and longitude) exerted a more pronounced influence (Jiang et al. 2024). Our results showed that sediment exhibited significantly higher alpha diversity than the water column in spring and summer, while the opposite trend was observed in autumn and winter. The warm season's elevated water temperatures may indirectly boost sediment alpha diversity through temperature‐mediated stimulation of benthic fish metabolism (e.g., *Cynoglossus*, *Platycephalus*), leading to greater eDNA shedding via mucus and excretion (Killen et al. 2010; Richard et al. 2017; Wood et al. 2020; Wang et al. 2023). Rainfall‐driven runoff during the same period is hypothesized to introduce substantial terrestrial organic matter, potentially promoting microbial degradation and the release of sediment‐bound eDNA (Barnes et al. 2014; Zhao et al. 2023). Furthermore, phytoplankton blooms may induce a biological pump effect (Turner 2015), facilitating the downward flux of eDNA from pelagic fish, thereby enriching the sedimentary eDNA pool. In contrast, during autumn‐winter, intensified vertical mixing potentially associated with the winter monsoon may contribute to the resuspension of sedimentary eDNA, enhancing the detectability of benthic species signals in the water column (Steinke et al. 2011). The increased RRA of *Cynoglossus* in winter water samples supports this inference. Moreover, lower water temperatures likely suppress fish behavioral/physiological activity, potentially leading to more localized eDNA retention and stronger spatial gradients in the water column (Lacoursière‐Roussel et al. 2016; Hansen et al. 2018), which could further contribute to the spatial heterogeneity observed in waterborne community structure. The community assembly mechanisms in Daya Bay's sediment‐water continuum habitat also exhibited significant seasonal variations. The fish community in Daya Bay demonstrated higher species richness in summer, which may reflect differential dispersal behaviors associated with reproductive periods, such as the selection strategies for various spawning grounds. In contrast, the fish community during the winter shows greater spatial differentiation, which could be linked to the migration of certain species to lower‐latitude, warmer waters for overwintering (Jiang et al. 2024). Notably, the elevated habitat beta diversity observed in winter is potentially consistent with the predictions of neutral theory, suggesting a hypothesis that intensified environmental filtering under low‐temperature and resource‐limited conditions might amplify the dominance of stochastic processes (including ecological drift and local extinction‐colonization dynamics) in community structuring (Rosindell et al. 2011; Hubbell 2001; Ford and Roberts 2020). Additionally, human disturbances, such as bottom trawl fisheries during the autumn and winter, may also exacerbate population fluctuations and reduce the stability of community structures (van Denderen et al. 2016; Ruan et al. 2022; Thomsen et al. 2016; Watson et al. 2013).

Using eDNA technology, this study identified core fish taxa that persist across seasons and habitats in the waters of Daya Bay, South China Sea—including genera such as *Photopectoralis*, *Konosirus*, and *Plotosus*—highlighting the structural stability and ecological adaptability of local fish communities. As a highly sensitive and non‐invasive biomonitoring tool, eDNA provided high‐resolution species detection, which enabled a deeper understanding of community dynamics (Sahu et al. 2023). However, certain technical limitations remain. For instance, we adopted a standardized rarefaction approach (5004 sequences per sample), which helps minimize sequencing depth–related biases in diversity analysis but may reduce the probability of detecting rare species. Future research could enhance rare or low RRA species detection by incorporating differential sequencing depth designs or adopting bioinformatics pipelines optimized for rare ASVs to improve the accuracy of biodiversity assessments (Xia et al. 2021). In addition, it is important to note that our taxonomic assignments were based on general reference databases. While this approach maximized taxonomic coverage and reduced the likelihood of missing species, it may have limited the accuracy and resolution of species identification. Using curated, region‐specific reference libraries would likely enhance classification confidence and allow more precise biodiversity assessments. Developing such curated databases should therefore be considered a priority for future studies in subtropical and tropical marine ecosystems.

It is also important to acknowledge that the interpretation of RRA in eDNA metabarcoding carries several inherent limitations. Relative Read Abundance does not necessarily correspond to organismal biomass or absolute population size, as amplification efficiency, primer–template mismatches, and species‐specific DNA shedding and degradation rates can all introduce distortion (Shelton et al. 2023; Rourke et al. 2022). Consequently, conclusions based solely on proportional data should be treated with caution. In this study, proportional read data were used only to compare broad‐scale community patterns across seasons and habitats, rather than to infer quantitative RRA. Future work could address these limitations more directly by incorporating mock community calibration to quantify and correct primer‐ and taxon‐specific amplification biases (Shelton et al. 2023; Guri et al. 2024). Furthermore, model‐based frameworks that explicitly account for amplification and sequencing biases, combined with the use of internal standards, may further enhance the robustness of RRA estimates. Collectively, these efforts will improve the interpretability of eDNA‐based community patterns and strengthen their utility for long‐term biodiversity monitoring and resource management (Rourke et al. 2022). Furthermore, it should be acknowledged that while our laboratory controls showed no evidence of contamination, the absence of field and filtration blanks represents a limitation of the current sampling design. Future eDNA monitoring in this region should incorporate a more comprehensive suite of controls to further enhance data transparency and mitigate risks of low‐level external contamination. Additionally, while the Random Forest model provided valuable insights into discriminative taxa, we acknowledge that without independent cross‐validation or stability selection, these results remain exploratory. Future studies should prioritize the integration of larger datasets for model validation to enhance the inferential robustness of machine‐learning‐based indicators.

Additionally, from an ecological management perspective, several species with clear seasonal indicators were identified in our work, including *Plotosus lineatus* , *Solea ovata* , *Nematalosa japonica* , and *Nuchequula nuchalis*. The seasonal fluctuations in their RRA not only reflect species‐specific ecological adaptations but could also serve as effective bioindicators for assessing the ecosystem health of Daya Bay. To enhance management effectiveness, a season–habitat coupling framework could be implemented in the future (Naidoo et al. 2006), with particular emphasis on reinforcing conservation measures during ecologically sensitive periods, such as the winter season (Zhang et al. 2024; Zhao et al. 2022). Incorporating these indicator species into dynamic monitoring frameworks, coupled with the integration of hydrological remote sensing data, would facilitate the development of species–environment response models (Rose et al. 2015; Carignan and Villard 2002). These models could quantify the interactive effects of anthropogenic and natural drivers, providing a scientific basis for evidence‐based adaptive management.

Despite the insights gained from this spatiotemporal assessment, several limitations of the current study must be acknowledged. First, the absence of concurrent, high‐resolution environmental monitoring data at each sampling station prevents the definitive attribution of observed community shifts to specific causal drivers. Consequently, the proposed mechanisms—such as monsoon‐driven resuspension, rainfall‐induced nutrient loading, and neutral theory filtering—should be positioned as plausible theoretical hypotheses rather than established causal relationships. These interpretations provide a valuable conceptual framework, but their validation requires further research integrating eDNA metabarcoding with real‐time hydrographic sensors and predictive environmental modeling. Future longitudinal monitoring efforts in Daya Bay should prioritize the simultaneous collection of physical and chemical covariates to rigorously test the ecological mechanisms proposed herein.

In conclusion, our work documents discernible ecological differentiation patterns and seasonal successional dynamics in Daya Bay fish communities across habitat gradients. These observations provide fundamental insights into niche partitioning mechanisms governing tropical–subtropical coastal fish assemblages. Continued implementation of eDNA monitoring in this region could enhance near‐real‐time ecosystem surveillance and early‐warning capabilities, thereby providing a rigorous scientific and technical foundation for adaptive management and the conservation of marine biodiversity.

## Author Contributions

**Shannan Xu:** conceptualization (equal), data curation (equal), formal analysis (equal), methodology (equal), project administration (equal), resources (equal), software (equal), validation (equal), writing – original draft (equal). **Yayuan Xiao:** conceptualization (equal), data curation (equal), formal analysis (equal), investigation (equal), project administration (equal), validation (equal), visualization (equal), writing – original draft (equal). **Min Li:** conceptualization (equal), resources (equal), software (equal), supervision (equal), writing – review and editing (equal). **Jiangtao Fan:** conceptualization (equal), investigation (equal), project administration (equal), visualization (equal), writing – original draft (equal). **Zuozhi Chen:** conceptualization (equal), funding acquisition (equal), resources (equal), supervision (equal), writing – review and editing (equal).

## Funding

This work was supported by the National Key R&D Program of China (2024YFD2400401), Financial Fund of the Ministry of Agriculture and Rural Affairs, P. R. China “Survey of offshore and open‐sea fishery resources in the South China Sea” (2021‐2025), Central Public‐interest Scientific Institution Basal Research Fund, CAFS (No. 2023TD16).

## Conflicts of Interest

The authors declare no conflicts of interest.

## Supporting information


**Figure S1:** Season‐ and habitat‐specific indicator taxa identified by the random forest model.
**Table S1:** Metadata of 233 environmental DNA (eDNA) samples collected seasonally in Daya Bay.

## Data Availability

The raw data were deposited in NCBI with the accession number PRJNA1268712 (https://www.ncbi.nlm.nih.gov/sra/?term=PRJNA1268712). In addition, the ASV taxonomy table (https://doi.org/10.5281/zenodo.17054007) and the ASV representative sequences (https://doi.org/10.5281/zenodo.17054170) have been deposited in Zenodo.
