# Supplementary material for: Integrating Water Column and Sediment eDNA Metabarcoding Reveals Seasonal Turnover and Habitat‐Specific Differentiation of Fish Communities in a Subtropical Coastal Bay
Source: Ecol Evol. 2026 Jul 9;16(7):e73988. doi: 10.1002/ece3.73988 (PMC13349660; doi:10.1002/ece3.73988)
Supplement: Supplementary file 1 — Figure S1: Season‐ and habitat‐specific indicator taxa identified by the random forest model. Table S1: Metadata of 233 environmental DNA (eDNA) samples collected seasonally in Daya Bay. [file ECE3-16-e73988-s001.docx]

# Supplementary

**Table S1** Metadata of 233 environmental DNA (eDNA) samples collected seasonally in Daya Bay

| **Number** | **SampleID** | **Season** | **Type** |
| --- | --- | --- | --- |
| 1 | AWS1-1 | Autumn | Water |
| 2 | AWS1-2 | Autumn | Water |
| 3 | AWS1-3 | Autumn | Water |
| 4 | AWS2-1 | Autumn | Water |
| 5 | AWS2-2 | Autumn | Water |
| 6 | AWS2-3 | Autumn | Water |
| 7 | AWS3-1 | Autumn | Water |
| 8 | AWS3-2 | Autumn | Water |
| 9 | AWS3-3 | Autumn | Water |
| 10 | AWS4-2 | Autumn | Water |
| 11 | AWS4-3 | Autumn | Water |
| 12 | AWS5-1 | Autumn | Water |
| 13 | AWS5-2 | Autumn | Water |
| 14 | AWS5-3 | Autumn | Water |
| 15 | AWS6-1 | Autumn | Water |
| 16 | AWS6-2 | Autumn | Water |
| 17 | AWS6-3 | Autumn | Water |
| 18 | AWS7-1 | Autumn | Water |
| 19 | AWS8-3 | Autumn | Water |
| 20 | AWS9-1 | Autumn | Water |
| 21 | AWS9-2 | Autumn | Water |
| 22 | AWS9-3 | Autumn | Water |
| 23 | AWS10-1 | Autumn | Water |
| 24 | AWS10-2 | Autumn | Water |
| 25 | AWS10-3 | Autumn | Water |
| 26 | AWS11-1 | Autumn | Water |
| 27 | AWS11-2 | Autumn | Water |
| 28 | AWS11-3 | Autumn | Water |
| 29 | AWS12-1 | Autumn | Water |
| 30 | AWS12-2 | Autumn | Water |
| 31 | AWS12-3 | Autumn | Water |
| 32 | AWS13-2 | Autumn | Water |
| 33 | AWS13-3 | Autumn | Water |
| 34 | AWS14-2 | Autumn | Water |
| 35 | AWS14-3 | Autumn | Water |
| 36 | WWS1-1 | Winter | Water |
| 37 | WWS1-2 | Winter | Water |
| 38 | WWS2-1 | Winter | Water |
| 39 | WWS3-1 | Winter | Water |
| 40 | WWS3-2 | Winter | Water |
| 41 | WWS4-1 | Winter | Water |
| 42 | WWS6-2 | Winter | Water |
| 43 | WWS7-1 | Winter | Water |
| 44 | WWS7-2 | Winter | Water |
| 45 | WWS8-2 | Winter | Water |
| 46 | WWS9-1 | Winter | Water |
| 47 | WWS11-1 | Winter | Water |
| 48 | WWS11-2 | Winter | Water |
| 49 | WWS11-3 | Winter | Water |
| 50 | WWS12-1 | Winter | Water |
| 51 | WWS12-2 | Winter | Water |
| 52 | WWS12-3 | Winter | Water |
| 53 | WWS13-1 | Winter | Water |
| 54 | WWS13-3 | Winter | Water |
| 55 | WWS14-2 | Winter | Water |
| 56 | AMS1-1 | Autumn | Mud |
| 57 | AMS1-2 | Autumn | Mud |
| 58 | AMS1-3 | Autumn | Mud |
| 59 | AMS2-2 | Autumn | Mud |
| 60 | AMS3-1 | Autumn | Mud |
| 61 | AMS3-2 | Autumn | Mud |
| 62 | AMS4-2 | Autumn | Mud |
| 63 | AMS4-3 | Autumn | Mud |
| 64 | AMS6-2 | Autumn | Mud |
| 65 | AMS7-2 | Autumn | Mud |
| 66 | AMS8-2 | Autumn | Mud |
| 67 | AMS8-3 | Autumn | Mud |
| 68 | AMS10-1 | Autumn | Mud |
| 69 | AMS10-2 | Autumn | Mud |
| 70 | AMS10-3 | Autumn | Mud |
| 71 | AMS11-1 | Autumn | Mud |
| 72 | AMS11-2 | Autumn | Mud |
| 73 | AMS11-3 | Autumn | Mud |
| 74 | AMS12-2 | Autumn | Mud |
| 75 | AMS13-2 | Autumn | Mud |
| 76 | AMS14-1 | Autumn | Mud |
| 77 | AMS14-2 | Autumn | Mud |
| 78 | WMS1-1 | Winter | Mud |
| 79 | WMS1-3 | Winter | Mud |
| 80 | WMS2-1 | Winter | Mud |
| 81 | WMS2-2 | Winter | Mud |
| 82 | WMS3-1 | Winter | Mud |
| 83 | WMS3-3 | Winter | Mud |
| 84 | WMS4-1 | Winter | Mud |
| 85 | WMS4-2 | Winter | Mud |
| 86 | WMS4-3 | Winter | Mud |
| 87 | WMS5-2 | Winter | Mud |
| 88 | WMS6-1 | Winter | Mud |
| 89 | WMS6-3 | Winter | Mud |
| 90 | WMS8-2 | Winter | Mud |
| 91 | WMS8-3 | Winter | Mud |
| 92 | WMS10-2 | Winter | Mud |
| 93 | WMS11-1 | Winter | Mud |
| 94 | WMS13-1 | Winter | Mud |
| 95 | WMS14-2 | Winter | Mud |
| 96 | SWS1-1 | Spring | Water |
| 97 | SWS1-2 | Spring | Water |
| 98 | SWS1-3 | Spring | Water |
| 99 | SWS2-2 | Spring | Water |
| 100 | SWS3-1 | Spring | Water |
| 101 | SWS3-3 | Spring | Water |
| 102 | SWS4-1 | Spring | Water |
| 103 | SWS5-1 | Spring | Water |
| 104 | SWS5-2 | Spring | Water |
| 105 | SWS6-1 | Spring | Water |
| 106 | SWS6-3 | Spring | Water |
| 107 | SWS7-1 | Spring | Water |
| 108 | SWS7-2 | Spring | Water |
| 109 | SWS7-3 | Spring | Water |
| 110 | SWS8-1 | Spring | Water |
| 111 | SWS8-2 | Spring | Water |
| 112 | SWS9-1 | Spring | Water |
| 113 | SWS9-2 | Spring | Water |
| 114 | SWS9-3 | Spring | Water |
| 115 | SWS10-1 | Spring | Water |
| 116 | SWS10-2 | Spring | Water |
| 117 | SWS10-3 | Spring | Water |
| 118 | SWS11-1 | Spring | Water |
| 119 | SWS11-2 | Spring | Water |
| 120 | SWS11-3 | Spring | Water |
| 121 | SWS12-1 | Spring | Water |
| 122 | SWS12-2 | Spring | Water |
| 123 | SWS12-3 | Spring | Water |
| 124 | SWS13-1 | Spring | Water |
| 125 | SWS13-2 | Spring | Water |
| 126 | SWS13-3 | Spring | Water |
| 127 | SWS14-1 | Spring | Water |
| 128 | SWS14-2 | Spring | Water |
| 129 | SWS14-3 | Spring | Water |
| 130 | SMS1-1 | Spring | Mud |
| 131 | SMS1-2 | Spring | Mud |
| 132 | SMS1-3 | Spring | Mud |
| 133 | SMS2-1 | Spring | Mud |
| 134 | SMS2-2 | Spring | Mud |
| 135 | SMS2-3 | Spring | Mud |
| 136 | SMS3-1 | Spring | Mud |
| 137 | SMS3-2 | Spring | Mud |
| 138 | SMS3-3 | Spring | Mud |
| 139 | SMS4-2 | Spring | Mud |
| 140 | SMS4-3 | Spring | Mud |
| 141 | SMS5-1 | Spring | Mud |
| 142 | SMS5-2 | Spring | Mud |
| 143 | SMS5-3 | Spring | Mud |
| 144 | SMS6-1 | Spring | Mud |
| 145 | SMS6-2 | Spring | Mud |
| 146 | SMS6-3 | Spring | Mud |
| 147 | SMS7-1 | Spring | Mud |
| 148 | SMS7-2 | Spring | Mud |
| 149 | SMS7-3 | Spring | Mud |
| 150 | SMS8-1 | Spring | Mud |
| 151 | SMS8-2 | Spring | Mud |
| 152 | SMS8-3 | Spring | Mud |
| 153 | SMS9-1 | Spring | Mud |
| 154 | SMS9-2 | Spring | Mud |
| 155 | SMS9-3 | Spring | Mud |
| 156 | SMS10-1 | Spring | Mud |
| 157 | SMS10-2 | Spring | Mud |
| 158 | SMS10-3 | Spring | Mud |
| 159 | SMS11-1 | Spring | Mud |
| 160 | SMS11-2 | Spring | Mud |
| 161 | SMS11-3 | Spring | Mud |
| 162 | SMS12-1 | Spring | Mud |
| 163 | SMS12-2 | Spring | Mud |
| 164 | SMS12-3 | Spring | Mud |
| 165 | SMS13-2 | Spring | Mud |
| 166 | SMS13-3 | Spring | Mud |
| 167 | SMS14-1 | Spring | Mud |
| 168 | SMS14-2 | Spring | Mud |
| 169 | SMS14-3 | Spring | Mud |
| 170 | UWS1-1 | Summer | Water |
| 171 | UWS2-1 | Summer | Water |
| 172 | UWS2-3 | Summer | Water |
| 173 | UWS3-2 | Summer | Water |
| 174 | UWS3-3 | Summer | Water |
| 175 | UWS4-1 | Summer | Water |
| 176 | UWS4-2 | Summer | Water |
| 177 | UWS4-3 | Summer | Water |
| 178 | UWS5-2 | Summer | Water |
| 179 | UWS5-3 | Summer | Water |
| 180 | UWS6-1 | Summer | Water |
| 181 | UWS7-1 | Summer | Water |
| 182 | UWS7-2 | Summer | Water |
| 183 | UWS8-1 | Summer | Water |
| 184 | UWS8-2 | Summer | Water |
| 185 | UWS8-3 | Summer | Water |
| 186 | UWS9-1 | Summer | Water |
| 187 | UWS9-2 | Summer | Water |
| 188 | UWS9-3 | Summer | Water |
| 189 | UWS10-1 | Summer | Water |
| 190 | UWS10-2 | Summer | Water |
| 191 | UWS10-3 | Summer | Water |
| 192 | UWS11-1 | Summer | Water |
| 193 | UWS11-2 | Summer | Water |
| 194 | UWS11-3 | Summer | Water |
| 195 | UWS12-2 | Summer | Water |
| 196 | UWS12-3 | Summer | Water |
| 197 | UWS13-1 | Summer | Water |
| 198 | UWS14-1 | Summer | Water |
| 199 | UWS14-2 | Summer | Water |
| 200 | UWS14-3 | Summer | Water |
| 201 | UMS1-1 | Summer | Mud |
| 202 | UMS1-2 | Summer | Mud |
| 203 | UMS1-3 | Summer | Mud |
| 204 | UMS2-1 | Summer | Mud |
| 205 | UMS2-2 | Summer | Mud |
| 206 | UMS2-3 | Summer | Mud |
| 207 | UMS3-1 | Summer | Mud |
| 208 | UMS3-2 | Summer | Mud |
| 209 | UMS3-3 | Summer | Mud |
| 210 | UMS4-1 | Summer | Mud |
| 211 | UMS4-3 | Summer | Mud |
| 212 | UMS5-2 | Summer | Mud |
| 213 | UMS6-1 | Summer | Mud |
| 214 | UMS6-2 | Summer | Mud |
| 215 | UMS6-3 | Summer | Mud |
| 216 | UMS7-2 | Summer | Mud |
| 217 | UMS7-3 | Summer | Mud |
| 218 | UMS8-1 | Summer | Mud |
| 219 | UMS8-2 | Summer | Mud |
| 220 | UMS8-3 | Summer | Mud |
| 221 | UMS9-1 | Summer | Mud |
| 222 | UMS9-2 | Summer | Mud |
| 223 | UMS9-3 | Summer | Mud |
| 224 | UMS10-1 | Summer | Mud |
| 225 | UMS10-2 | Summer | Mud |
| 226 | UMS10-3 | Summer | Mud |
| 227 | UMS11-1 | Summer | Mud |
| 228 | UMS12-1 | Summer | Mud |
| 229 | UMS12-3 | Summer | Mud |
| 230 | UMS13-1 | Summer | Mud |
| 231 | UMS13-2 | Summer | Mud |
| 232 | UMS13-3 | Summer | Mud |
| 233 | UMS14-2 | Summer | Mud |

**Figure S1.** Season- and habitat-specific indicator taxa identified by the random forest model. (A) autumn, (B) winter, (C) spring, and (D) summer. * *p* < 0.05, ** *p* <0 .01, *** *p* < 0.001, **** *p* < 0.0001.
